# Role of Surface Chemistry in the In Vitro Lung Response to Nanofibrillated Cellulose

**DOI:** 10.3390/nano11020389

**Published:** 2021-02-03

**Authors:** Kukka Aimonen, Satu Suhonen, Mira Hartikainen, Viviana R. Lopes, Hannu Norppa, Natalia Ferraz, Julia Catalán

**Affiliations:** 1 Finnish Institute of Occupational Health, Box 40, Työterveyslaitos, 00032 Helsinki, Finland; kukka.aimonen@ttl.fi (K.A.); satu.suhonen@ttl.fi (S.S.); mira.hartikainen@ttl.fi (M.H.); hannu.norppa@ttl.fi (H.N.); 2 Nanotechnology and Functional Materials, Department of Materials Science and Engineering, Uppsala University, Box 35, 751 03 Uppsala, Sweden; vivianarlopes@gmail.com (V.R.L.); natalia.ferraz@angstrom.uu.se (N.F.); 3 Department of Anatomy, Embryology and Genetics, University of Zaragoza, 50013 Zaragoza, Spain

**Keywords:** nanofibrillated cellulose, surface chemistry, genotoxicity, nanotoxicity, nanocellulose, reactive oxygen species, human bronchial epithelial cells

## Abstract

Wood-derived nanofibrillated cellulose (NFC) has emerged as a sustainable material with a wide range of applications and increasing presence in the market. Surface charges are introduced during the preparation of NFC to facilitate the defibrillation process, which may also alter the toxicological properties of NFC. In the present study, we examined the in vitro toxicity of NFCs with five surface chemistries: nonfunctionalized, carboxymethylated, phosphorylated, sulfoethylated, and hydroxypropyltrimethylammonium-substituted. The NFC samples were characterized for surface functional group density, surface charge, and fiber morphology. Fibril aggregates predominated in the nonfunctionalized NFC, while individual nanofibrils were observed in the functionalized NFCs. Differences in surface group density among the functionalized NFCs were reflected in the fiber thickness of these samples. In human bronchial epithelial (BEAS-2B) cells, all NFCs showed low cytotoxicity (CellTiter-GloVR luminescent cell viability assay) which never exceeded 10% at any exposure time. None of the NFCs induced genotoxic effects, as evaluated by the alkaline comet assay and the cytokinesis-block micronucleus assay. The nonfunctionalized and carboxymethylated NFCs were able to increase intracellular reactive oxygen species (ROS) formation (chloromethyl derivative of 2′,7′-dichlorodihydrofluorescein diacetate assay). However, ROS induction did not result in increased DNA or chromosome damage.

## 1. Introduction

Cellulose nanofibers have emerged as sustainable and environmentally friendly materials with a wide range of industrial and medical applications [1]. There are several types of nanocelluloses that can be obtained from different raw materials, the most common sources being hard- and softwood chemical pulp fibers [2]. Wood pulp fibers are processed with chemical and enzymatic pretreatments to facilitate the structural deconstruction of the fibers into two main types of nanocelluloses: nanofibrillated cellulose (NFC) and cellulose nanocrystal [2,3]. Wood NFC is composed of amorphous and crystalline domains [4] and is characterized by having a high-aspect ratio, with a typical diameter of 2–10 nm and length in the micrometer-scale (>1 µm) [5,6]. NCFs are obtained from wood pulp fibers by mechanical fibrillation, which can be performed by using, e.g., homogenizers, fluidizers and grinders [7]. Prior to the mechanical fibrillation, various chemical and enzymatic pretreatments can be applied to ease the fibrillation of fibers into homogeneous nanofibril dispersions. Chemical pretreatments introduce for example carboxyl, carboxymethyl, and aldehyde groups or phosphoryl side groups on the surface of NFC [8]. Such pretreatments affect not only the surface chemistry of the nanofibrils but also properties like fiber dimensions, specific surface area, and degree of branching of the nanofibrils [9].

The increasing use of nanocelluloses in multiple applications should be accompanied by an adequate assessment of their safety—especially in occupational settings, where inhalation is considered the primary route of exposure [10]. A life cycle risk assessment of nanocelluloses identified inhalation of dry nanocellulose powders or, in the case of wet slurry, airborne nanocellulose-containing droplets, as the most relevant exposure scenarios during the production and manufacturing of nanocelluloses [11].

Due to their natural origin, cellulosic materials are often assumed not to be toxic [10]. However, the long pulmonary biopersistence of these materials [12,13,14,15], together with the high aspect ratio, raise concerns about potential effects on human health, especially if inhaled [10]. Furthermore, some features of NFCs, such as nanometer size, large surface area, and modified surface chemistry, may impart novel material properties and biological behavior, as compared with macro-scale materials [16]. Therefore, it is necessary to address the human health and environmental safety aspects of nanocelluloses before scaling up their production.

There is still scarce knowledge on the potential adverse health effects of NFCs, despite the increasing number of studies performed during the last few years. As summarized in different reviews on this topic [17,18,19,20,21], toxicological studies show contradictory findings. The conflicting results may partly be due to variation among NFCs because of different factors, e.g., cellulose source, mechanical fibrillation procedure, or pretreatments [21], which can modify the material properties. It is well-recognized that the physicochemical features of nanomaterials may affect their toxicity [16,22,23], surface chemistry being one of the most relevant ones [18]. Surface modifications can impart new beneficial properties to nanocelluloses, increasing their applicability in, e.g., healthcare products and food packaging [24]. However, different functionalization will determine differences in the agglomeration rate, hydrophobicity, surface charge, and surface chemistry of nanocelluloses, which may affect their cellular uptake, interaction with subcellular organelles, and downstream biological responses [19]. Surface chemistry was reported to drive in vitro inflammatory response to NFC [25], while no differences in cell metabolic activity or cell membrane integrity were observed when diverse in vitro cell models where exposed to differently functionalized NFC materials [14,24,25].

Studies addressing the genotoxic potential of NCF are too scarce to allow clear conclusions [2,19]. Genotoxic effects were observed in mouse 3T3 fibroblasts exposed to curauá- and brown cotton-derived NFC [26]. Ventura et al. [27] also reported an increase in chromosome damage, but not DNA damage, in human lung epithelial alveolar A549 cells cocultured with THP-1 macrophages after being treated with a TEMPO (2,2,6,6-tetramethyl-piperidin-1-oxyl) oxidized NFC. Furthermore, genotoxic effects were observed in the lung tissue of mice exposed to an enzymatically pretreated NFC [13,15] and to a TEMPO oxidized NFC [28]. On the other hand, no DNA or chromosome damage was induced by unmodified and enzymatically or chemically pretreated NFC on human bronchial epithelial BEAS-2B cells [13]. However, none of these studies compared NFC derived from the same source with different surface chemistry.

In the present study, we investigated the role of surface chemistry in modulating the in vitro toxic potential of NFC, by analyzing the ability of the materials to induce cytotoxic effects, the formation of reactive oxygen species (ROS), and DNA and chromosomal damage in human bronchial epithelial BEAS-2B cells. Additionally, fluorescent staining of the nanofibrils was used to assess the cellular uptake of NFC. Four NFC materials with various surface functionalization (carboxymethylation, hydroxypropyltrimethylammonium substitution, phosphorylation, and sulfoethylation), together with nonfunctionalized NFC, were included in this study. While other studies have investigated the influence of surface modified NFC on several toxicological endpoints using different cell models, to the best of our knowledge, this is the first study investigating the genotoxic response to NFCs that only differ in their surface chemistry.

## 2. Materials and Methods

### 2.1. Synthesis and Surface Modification of the NFC Materials

The NFC materials were provided by RISE Bioeconomy (Stockholm, Sweden) and were produced from commercial never-dried bleached sulfite softwood dissolving pulp (Domsjö Fabriker AB, Sweden). Nonfunctionalized NFC, herein referred as unmodified (U-NFC), was produced by enzymatic pretreatment of the wood pulp following the protocol presented by Pääkoo et al. [29]. Carboxymethylated NFC (C-NFC) and hydroxypropyltrimethylammonium NFC (H-NFC) were prepared as described by Hua et al. [30]. Phosphorylated NFC (P-NFC) was produced following the method described by Naderi et al. [31], using a 4:1 phosphorous:glucopyranose molar ratio, followed by 5 microfluidizer passes at 1700 bar. To prepare sulfoethylated NFC (S-NFC), the protocol described by Naderi et al. [32] was followed. The surface functional group density of all NFC samples was determined as described in Lopes et al. [24].

**Figure 1:**
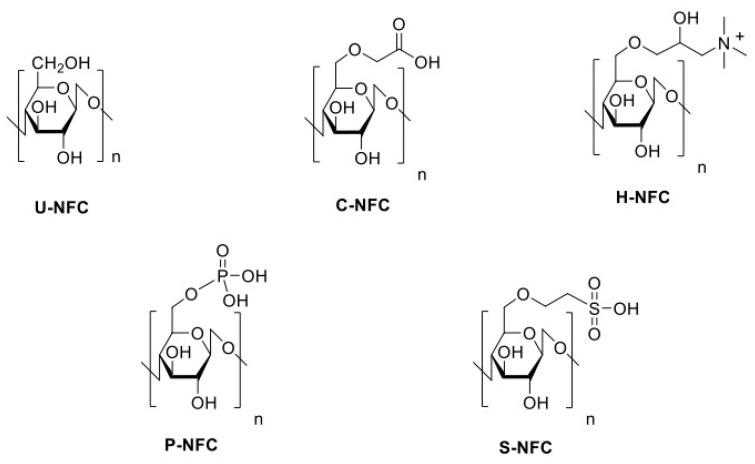
Chemical structures of the nanofibrillated cellulose (NFC) materials under study.

The chemical structures of the NFC materials are shown in Figure 1.

### 2.2. Preparation of the NFC Exposure Suspensions

The stock suspensions of the NFCs were prepared in phosphate buffer (PBS) at 5 mg/mL and dispersed as previously described [25]. Briefly, the stock suspensions were dispersed using a Branson Sonifier 450D (400 W, 60 Hz; Danbury, CT, USA) with a 13-mm horn for 12 min. The suspensions were then sterilized by autoclaving, except for H-NFC which was subject to ultraviolet radiation (UV) treatment during two cycles of 45 min each. Then, the stock suspensions were diluted in cell culture medium and sonicated for 30 min in a 37 kHz Elmasonic S15H ultrasonic bath cleaner (Elma Schmidbauer GmbH, Singen, Germany) before being added to the cells.

### 2.3. Characterization of the NFC Materials

#### 2.3.1. Bacterial Contamination, Endotoxin and (1,3)-β-D-Glucan Levels

Bacterial contamination was tested using the 3M™ Petrifilm™ Aerobic Count Plates, whereas bacterial lipopolysaccharide content (endotoxin level) was measured using the Pierce^TM^ LAL Chromogenic Endotoxin Quantitation Kit (Thermo Fisher Scientific, Waltham, MA, USA), following the instructions provided by the manufacturer. Prior to the test, the samples were heated at 75 °C for 15 min, to promote the release of endotoxins from the material.

The level of (1,3)-β-D-glucans in the NFC samples was investigated using the (1,3)-β-D-glucan detection kit Glucantell with diazo-reagents for endpoint assay (Associates of Cape Cod Inc., Liverpool, UK), according to the protocol described by the manufacturer. The analyzed samples were extracts obtained after incubating the NFC suspensions at 75 °C for 15 min.

#### 2.3.2. Fiber Morphology

Transmission electron microscopy (TEM) imaging was used to investigate the morphology of the fibers. Samples were prepared as described by Usov et al. [33]. Briefly, 5 μL of NFC suspension (0.1% in deionized water, dispersed through ultrasonication as described in Section 2.2) were deposited onto copper TEM grids with formvar carbon support film for 1 min. Thereafter, the sample grids were stained by adding 5 μL of 2% uranyl acetate for 1 s and again 5 μL of 5% uranyl acetate for 15 s. After each step, the excess of moisture was drained along the periphery using filter paper. The grids were examined using a FEI Titan Themis TEM (Thermofisher Scientific, Waltham, MA, USA) operated at 200 kV.

#### 2.3.3. Zeta-Potential

The z-potential of the NFC materials was determined in 10 mM NaCl and in cell culture medium (serum-free LHC-9 medium). The measurements in 10 mM NaCl were done following the protocol described by Lopes et al. [24]. For the measurements in cell culture medium, z-potential was determined as an average of three separate measurements with Zetasizer Nano ZS (Malvern Instruments Ltd., Malvern, UK). Dispersions of 0.001% (*w*/*w*) of the NFC were prepared in cell culture medium, following the same dispersion protocol as used for the in vitro exposures. The measurements were conducted at 37 °C in folded capillary cell directly after dispersing the fibers.

### 2.4. Cell Culture

Transformed human bronchial epithelial BEAS-2B cells, exhibiting an epithelial phenotype [34], were obtained from the American Type Culture Collection through LGC Promochem AB (Borås, Sweden). The BEAS-2B cells were grown in serum-free LHC-9 medium (Gibco, Life Technologies Corporation, Grand Island, NY, USA) at 37 °C in a humidified atmosphere of 5% CO_2_. Log-phase BEAS-2B cells were plated on 48-well plates (comet assay), 96-well plates (cytotoxicity and ROS assays) and 2-well chamber slides (micronucleus assay) from one to three days prior to exposure to the NFC materials.

### 2.5. Cellular Internalization of NFC

To assess the potential internalization of NFC by BEAS-2B cells, NFC was stained with the Calcofluor White Stain (Merck KGaA, Darmstadt, Germany). The staining was performed on the same slides that were used for the scoring of the micronucleus frequency (for slide preparation, see below). At harvest, the cells were treated for 15 min at 25 °C with cellulase (8.7 µL/mL, Cellic CTec2, Novozymes, Bagesvaerd, Denmark) to remove excess NFC outside the cells. The procedure of the calcofluor staining has previously been described [35]. In brief, a drop of calcofluor was applied onto acridine orange-stained slides and incubated under a cover slip for 1 min. The sample was thereafter examined with a fluorescence microscope (ZEISS Axio Imager Z1, Carl Zeiss AG, Oberkochen, Germany), using DAPI/FITC/TRITC triple filter and 40x objective lens.

### 2.6. Cytotoxicity Assessment

BEAS-2B cells were seeded in white clear flat bottom 96-well plates (Corning, NY, USA) at a density of 2 × 10^4^ cells/well (200 µL/well; culture area 0.32 cm^2^/well) and grown to semiconfluency, after which they were exposed to NFC dispersions for 24 and 48 h at eight doses: 3.9, 7.8, 15.6, 31.2, 62.5, 125, 250, and 500 µg/mL (equivalent to 2.4, 4.9, 9.8, 19.5, 39.1, 78.1, 156.2, and 312.5 µg/cm^2^). 0.1% Triton X-100 (AppliChem, Darmstadt, Germany) was used as positive control, while untreated cells served as negative control at each time point. All treatments were performed in quadruplicate, and the experiments were repeated three times.

Cytotoxicity was measured using the CellTiter-GloVR Luminescent Cell Viability Assay (Promega, Madison, WI, USA) according to instructions provided by the manufacturer (Technical bulletin, Promega Corporation, revised 6/09, part# TB288). This assay estimates the number of metabolically active viable cells in the culture, based on the quantization of ATP, using Ultra-Glo^TM^ Recombinant Luciferase and measurement of relative luminescence by Fluoroskan Ascent FL (Thermo Electron Corporation, Vantaa, Finland). Because some nanomaterials have been described to have optical interference [36], the measurements also included the cellulose materials without cells. However, the NFCs showed no interference with the luminometric determinations.

Cytotoxicity was expressed as relative luminescence in the treated cultures in comparison with the untreated cultures. This assay reflects all treatment-related effects (necrosis, cell cycle delay and apoptosis) that reduce the number of viable or living cells. In addition, as requested by the OECD TG 487 [37], cytostasis was always measured when performing the micronucleus assay, as a way of assessing cytotoxicity in the same cultures that were used for the micronucleus assay. In this way, the adequacy of the chosen dose range based on the luminometric assay could be confirmed.

### 2.7. Formation of Intracellular Reactive Oxygen Species (ROS)

The levels of intracellular ROS were measured using the chloromethyl derivative of 2′,7′-dichlorodihydrofluorescein diacetate (CM-H_2_DCFDA) (Invitrogen, Eugene, OR, USA), according to the manufacturer’s guidelines. DCFDA is a lipophilic cell permeable compound that is deacetylated in the cytoplasm by cellular esterases and later oxidized by ROS to a highly fluorescent molecule [25]. Derivatives with a thiol-reactive chloromethyl group allow for covalent binding to intracellular components, permitting even longer retention within the cell.

BEAS-2B cells were plated on black clear flat bottom 96-well plates (Corning, NY, USA) at a density of 20,000 cells/well (200 µL/well; culture area 0.32 cm^2^/well) and grown to semiconfluency for two days. After being washed with Gibco™ Dulbecco’s Phosphate-Buffered Saline (DPBS, Thermo Fisher Scientific, Paisley, Scotland), the cells were loaded with 2.5 µM CM-DCFDA in PBS for 30 min at 37 °C. Thereafter, the loading buffer was removed, and the cells were washed with PBS. Then the cells were treated with the NFC dispersions at eight doses: 3.9, 7.8, 15.6, 31.2, 62.5, 125, 250 and 500 µg/mL (equivalent to 2.4, 4.9, 9.8, 19.5, 39.1, 78.1, 156.2 and 312.5 µg/cm^2^). 2 mM H_2_O_2_ (Sigma-Aldrich Chemie, Steinheim, Germany) was used as a positive control, while untreated cells served as a negative control at each time point. Fluorescence was recorded at 3, 6, and 24 h (excitation 485 nm, emission 538 nm) using a plate reader (Fluoroskan Ascent FL, Vantaa, Finland). The average fluorescent intensity was calculated by subtracting background values. All treatments were performed in quadruplicates, and the experiments were repeated three times.

### 2.8. Genotoxicity Assessment

#### 2.8.1. Comet Assay

The comet (single cell gel electrophoresis) assay was used to study DNA strand breaks and alkaline labile sites in BEAS-2B cells after exposure to the NFC materials. BEAS-2B cells in log phase were plated in 48-well plates (culture area 0.95 cm^2^/well, culture medium volume 250 µL/well; Corning, NY, USA) two days prior to exposure. Exposure time was 24 h, and the cells were exposed to 6, 19, 56, 167, and 500 µg/mL (corresponding to 1.6, 5.0, 14.7, 43.9, and 131.6 µg/cm^2^) of each NFC. Untreated controls and positive controls treated with hydrogen peroxide (20 mM, Riedel-de Haen, Seelze, Germany) were included in all series. All treatments were performed in duplicate, and the experiments were repeated twice.

The comet assay was performed in alkaline conditions (pH > 13) as described previously [38]. The slides were coded, and one scorer performed the comet analysis using a fluorescence microscope (Axioplan 2, Zeiss, Jena, Germany) and an interactive automated comet counter (Komet 5.5, Kinetic Imaging Ltd., Liverpool, UK). The percentage of DNA in the comet tail from 200 cells per dose and experiment (two replicates per dose, two slides per replicate, 50 cells/slide) was used as a measure of the amount of DNA damage.

#### 2.8.2. Cytokinesis-Block Micronucleus Assay

The cytokinesis-block micronucleus assay was applied to study chromosomal damage in BEAS-2B cells after exposure to the NFCs. The cells were plated on 2-well chamber slides (culture area 4.2 cm^2^/well, culture medium volume 2 mL/well; Nunc, Roskilde, Denmark) at a density of 300,000 cells per well and incubated for 24 h, to reach semiconfluency, prior to the treatment.

Based on the cytotoxicity assay, the cells were exposed for 48 h to five doses of the dispersed materials: 6, 19, 56, 167, and 500 µg/mL (the corresponding doses were 2.8, 9.0, 26.7, 79.5, and 238.1 µg/cm^2^). Cytochalasin B (9 µg/mL; Sigma-Aldrich Chemie, Steinheim, Germany) was added to the cell cultures 6 h after starting the treatment, to induce binucleation of dividing cells. Untreated cultures and cultures treated with the positive control mitomycin C (MMC; Sigma-Aldrich, Steinheim, Germany; 150 ng/mL) were included in all experiments. All cultures were prepared in duplicate.

After the exposure, the cells were briefly rinsed in PBS, treated for 15 min at 25 °C with cellulase enzyme blend (8.7 µL/mL), and rinsed again with PBS. Cellulase treatment was performed to get rid of the noninternalized nanofibrils that could interfere with the micronucleus scoring. The slides were air-dried for 2 h, fixed for 10 min in absolute methanol at 25 °C, air-dried, and kept at −20 °C until staining. The cells were stained with acridine orange (32 mg/mL) for 1 min after which the slides were rinsed three times in Sörensen buffer (pH 6.8), stained with 4,6-diamidino-2-phenylindole (DAPI, 1 µg/mL) for 5 min, rinsed in tap water, and allowed to dry. The slides were kept at 4 °C protected from light. Immediately before analysis, the slides were mounted in Sörensen buffer and covered with a coverslip.

The slides were coded for a blinded analysis. All analyses were performed by one scorer. Cell proliferation was first measured to assess the possible effect of the treatments on cell cycle delay (cytostatic effect), as a means to ensure that the treatments were conducted at appropriate levels of cytotoxicity. To this end, cytostasis, based on the cytokinesis-blocked proliferation index (CBPI; OECD 2016), was calculated from 100 cells per replicate culture (200 cells per dose) as follows:

%Cytostasis = 100 − 100[(CBPI_treated_ − 1)/(CBPI_control_ − 1)]

where: CBPI = [(No. mononucleate cells) + 2(No. binucleate cells) + 3(No. multinucleate cells)]/(total No. cells).

To evaluate the frequency of micronucleated cells, micronuclei were scored in 4000 binucleate cells per treatment (2000 binucleate cells per replicate; two replicates per treatment) using ZEISS Axio Imager Z1 microscope (Carl Zeiss AG, Oberkochen, Germany). Binucleate cells and micronuclei in them were identified using a 40× objective lens using a FITC/TRITC double filter for acridine orange, and the micronuclei were verified with DAPI.

### 2.9. Statistical Analyses

One-way analysis of variance (ANOVA), followed by Dunnett’s multiple comparison post hoc test, was used to assess the effect of the dose in all the assays. Dose-response in the formation of ROS and in the comet and micronucleus assays was evaluated by linear regression analysis. Comparisons between the positive and negative control groups were performed by unpaired one-tailed *t*-test for all the assays. All analyses were performed using GraphPad Prism 8, version 8.3.1 (GraphPad Software, San Diego, CA, USA). The effects were considered significant if *p* < 0.05.

## 3. Results

### 3.1. Characterization of the NFCs

Table 1 summarizes the properties of the NFC samples under study. P-NFC had the highest surface group density among the functionalized materials, followed by H-NFC, S-NFC, and C-NFC. Low levels of carboxyl groups were quantified in U-NFC, which can be attributed to the presence of residual hemicellulose.

Z-potential measurements of the NFC suspensions in 10 mM NaCl reflected the presence of the surface charged groups, with positive values for the NFC material with the hydropropyltrimethylammonium substitution (H-NFC) and negative values for the C-NFC, P-NFC, and S-NFC samples. The differences in surface group density were reflected in the z-potential absolute values, with the highest degree of functionalization (P-NFC) corresponding to the highest z-potential value. The z-potential values obtained for the suspensions in cell culture medium did not significantly differ from the values obtained with the NaCl suspensions. The absence of proteins in the cell culture media and the similar pH compared with the NaCl suspensions explain this similarity.

**Figure 2:**
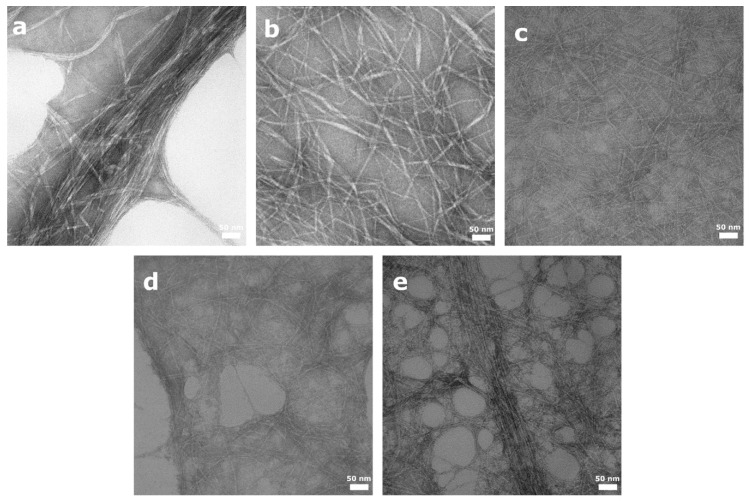
Representative TEM images of nanofibrillated cellulose (NFC) aqueous suspensions showing the morphology of the nanofibers. (**a**) U-NFC, (**b**) C-NFC, (**c**) H-NFC, (**d**) P-NFC and (**e**) S-NFC.

The morphology of the NFC materials can be observed in the TEM images displayed in Figure 2. As expected, due to the lack of surface charged groups in U-NFC (only low levels of residual carboxy groups were detected), the fibers formed aggregates (10–30 nm in diameter; Figure 2a), while the functionalization of the NFC materials resulted in better dispersion of the individual fibers. However, some differences were found between the functionalized materials, where the density of the surface groups seemed to influence the fiber morphology. H-NFC and P-NFC (Figure 2c,d, respectively) presented individual fibrils (4–5 nm in diameter), while C-NFC with the lowest surface group density of all functionalized materials showed fiber aggregates with thickness up to 15 nm (Figure 2b). Slight fiber aggregation was observed in the S-NFC sample (Figure 2e), which may also be related to its slightly lower surface group density compared with the highly defribrillated H-NFC and P-NFC.

No bacterial contamination was found in the dispersed NFC suspensions after being sterilized by autoclaving or UV treatment.

The results from the LAL assay showed that the NFC materials, except H-NFC, had a high level of endotoxins, which was above the upper detection limit (>1.2 EU/mL) of the assay. H-NFC showed a level of 0.12 EU/mL which was below the 0.5 EU/mL limit value established by the US Food Drug Agency for inhalation studies [39]. No test kit interference was detected with any of the materials. However, the potential contribution of β-glucan contaminants in the NFC samples to the observed endotoxin levels could not be dismissed, since the endotoxin kit employed did not inhibit the reaction with β-glucans.

When the presence of (1,3)-β-D-glucans in extracts of NFC was measured, values in the concentration range 250–20 pg/mL were found (Figure S1), thus indicating low levels of (1,3)-β-D-glucan contaminants in the NFC materials [40].

### 3.2. Internalization of NFCs

**Figure 3:**
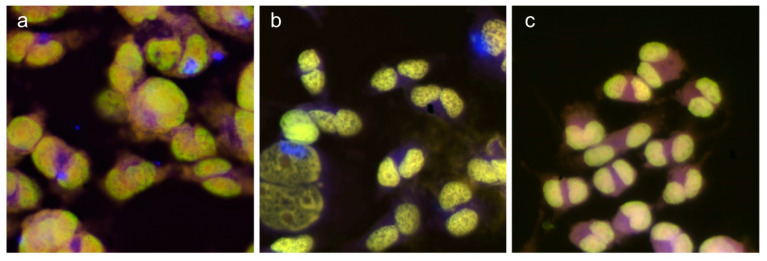
Examples of calcofluor staining (blue) in cells treated with U-NFC (167 µg/mL) (**a**), H-NFC (56 µg/mL) (**b**) and untreated cells (**c**). Counterstaining by acridine orange.

As calcofluor staining was performed on cellulase pretreated slides, noninternalized NFC was not expected to be present, and hence detected by the staining, in these preparations. Therefore, stained NFC that appeared associated with some cells (Figure 3a,b) may have reflected cellular internalization. However, it may also have been due to NFC material attached to the cell membrane that was not efficiently eliminated by the cellulase treatment. Nevertheless, most cells did not show calcofluor-stained material, suggesting that the possible NFC internalization concerned a minority of the cells. No calcofluor-stained material could be found in the untreated cultures (Figure 3c). As NFC on the cells could not reliably be distinguished from internalized NFC, it remains unclear how much the NFCs were actually taken up.

### 3.3. Cytotoxicity

**Figure 4:**
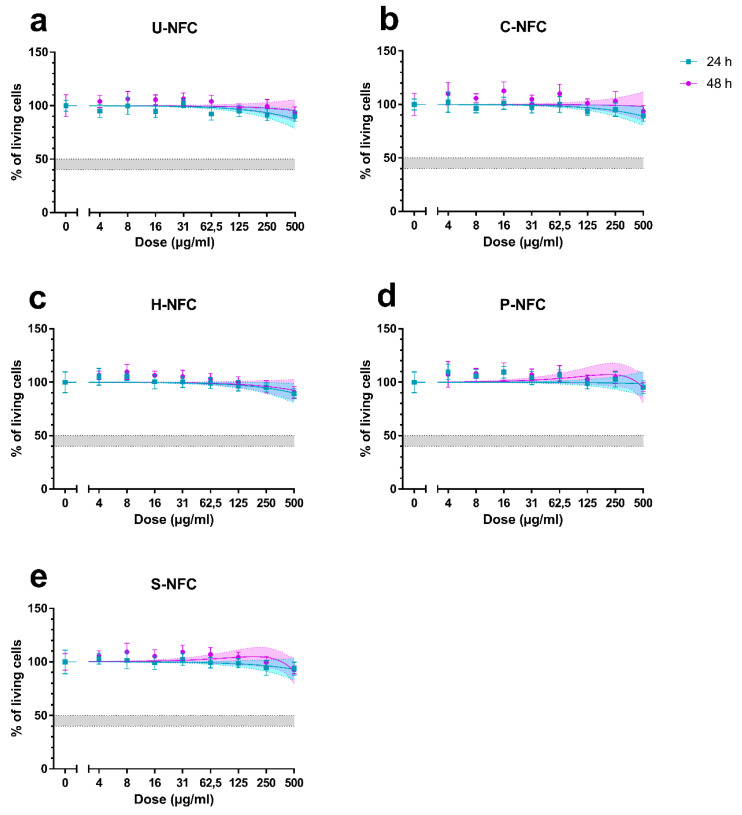
Cytotoxicity of the nanofibrillated cellulose (NFC) materials. The total number of living cells was counted at 24 and 48 h exposure to (**a**) U-NFC, (**b**) C-NFC, (**c**) H-NFC, (**d**) P-NFC and (**e**) S-NFC, and the results were normalized with the unexposed control. Results are presented as the mean ± standard error of the mean. Graphs are plotted with 95% confidence interval (colored areas). The range of 45 ± 5% living cells (corresponding to the 55 ± 5% cytotoxicity) is indicated by the grey area in each graph.

None of the tested NFCs significantly reduced the number of living cells present in the culture as compared with the negative control (Figure 4).

The purpose of the cytotoxicity assay was also to choose the range of doses of each NFC to be tested in the genotoxicity assays. According to OECD guidelines on the in vitro micronucleus test [37], the highest test substance concentration to be included in the assay (in the absence of micronucleus induction at earlier doses) should produce 55 ± 5% cytotoxicity. Higher levels may induce chromosome damage as a secondary effect of cytotoxicity [41] and should, therefore, be avoided. In the present study, the highest tested dose (500 µg/mL) did not reach the cytotoxicity limit for any of the NFCs (Figure 4). On the other hand, more concentrated dispersions were not stable enough. Hence, 500 µg/mL was chosen as the highest dose to be tested in the other assays.

The positive control, 0.1% Triton X-100, induced a statistically significant decrease in the number of living cells in all the experiments at both 24 h (3.31 ± 0.14% living cells in comparison with the negative control, *p* < 0.001) and 48 h (1.73 ± 0.06% living cells in comparison with the negative control, *p*< 0.001) exposure, confirming the validity of the experiments.

### 3.4. Induction of Intracellular ROS

**Figure 5:**
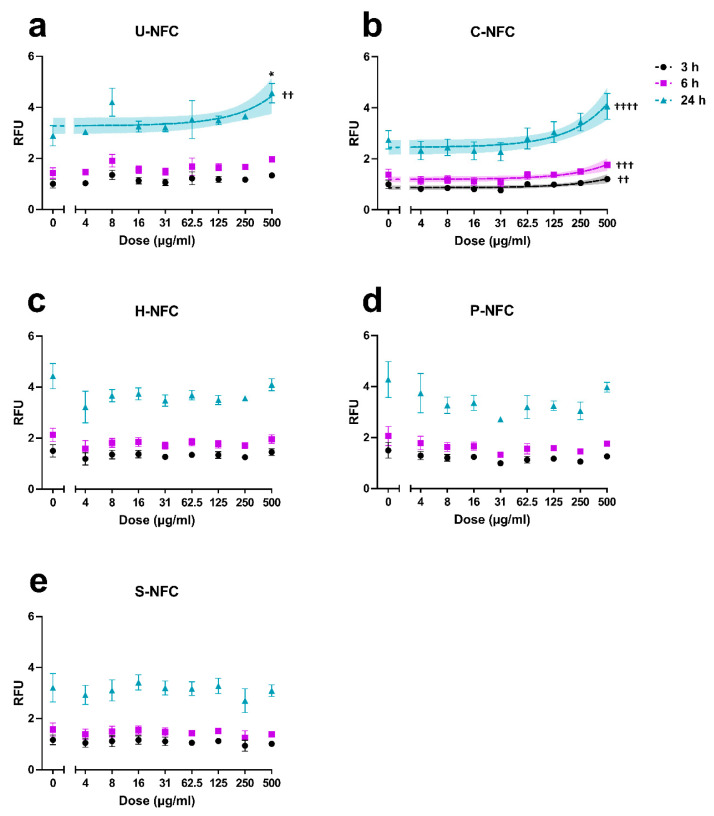
Induction of intracellular reactive oxygen species (ROS) by the nanofibrillated cellulose (NFC) materials. The production of ROS was assessed at 3, 6, and 24 h exposure to (**a**) U-NFC, (**b**) C-NFC, (**c**) H-NFC, (**d**) P-NFC and (**e**) S-NFC. Data are expressed as relative fluorescence units (RFU) and presented as the mean ± standard error of the mean. Significant linear dose-responses are plotted with 95% confidence interval (colored areas). Statistical significance in comparison with control cultures (one-way ANOVA): * *p* < 0.05. Statistical significance, linear regression: ^††^ *p* < 0.01; ^†††^ *p* < 0.001; ^††††^ *p* < 0.0001.

Figure 5 shows the induction of intracellular ROS at three different times of exposure (3, 6, and 24 h) to each of the NFCs. U-NFC induced a significant increase in ROS formation in comparison with the negative control at 500 µg/mL in the 24 h exposure (*p* = 0.0327). In addition, there was a significant linear dose-response (*p* = 0.0078) at this time point but not at earlier ones (Figure 5a). C-NFC did not induce a statistically significant increase in ROS formation at any dose or time point. However, there was a significant linear dose-response at 3 h (*p* = 0.0023), 6 h (*p* = 0.0004), and 24 h (*p* < 0.0001) of C-NFC exposure (Figure 5b).

H-NFC, P-NFC, and S-NFC induced neither a significant increase in ROS formation nor a significant linear dose-response, at any dose or exposure time (Figure 5c–e, respectively). The positive control, H_2_O_2_ (2 mM), statistically significantly increased ROS production over the negative control values in all the experiments performed at 3 h (8.12 ± 0.25-fold increase; *p* < 0.05), 6 h (6.87 ± 0.53-fold increase; *p* < 0.01) and 24 h (3.58 ± 0.07-fold increase; *p* < 0.05) exposure, confirming the validity of the experiments.

### 3.5. Genotoxicity

**Figure 6:**
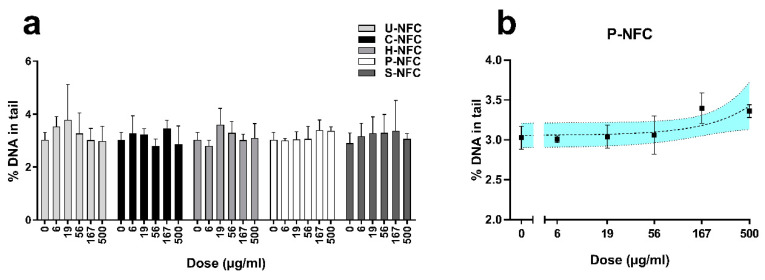
Induction of DNA damage by the nanofibrillated cellulose (NFC) materials. DNA strand breaks were assessed at 24 h exposure to (**a**) U-NFC, C-NFC, H-NFC, P-NFC, and S-NFC. A significant linear dose response (*p* = 0.0427) was induced by P-NFC (**b**). Data are expressed as percentage of DNA in tail and presented as the mean ± standard error of the mean. Significant linear dose-response is plotted with 95% confidence interval (colored area).

None of the NFC materials were able to induce an increase in DNA damage compared with the negative control at any of the tested doses (Figure 6a). A significant linear dose-response (*p* = 0.04) was only found for P-NFC (Figure 6b).

The positive control, H_2_O_2_ (20 mM), induced a statistically significant increase in the percentage of DNA in tail over the negative control values in all the experiments performed (19.43 ± 2.06-fold increase; *p* < 0.0001), confirming the validity of the experiments.

**Figure 7:**
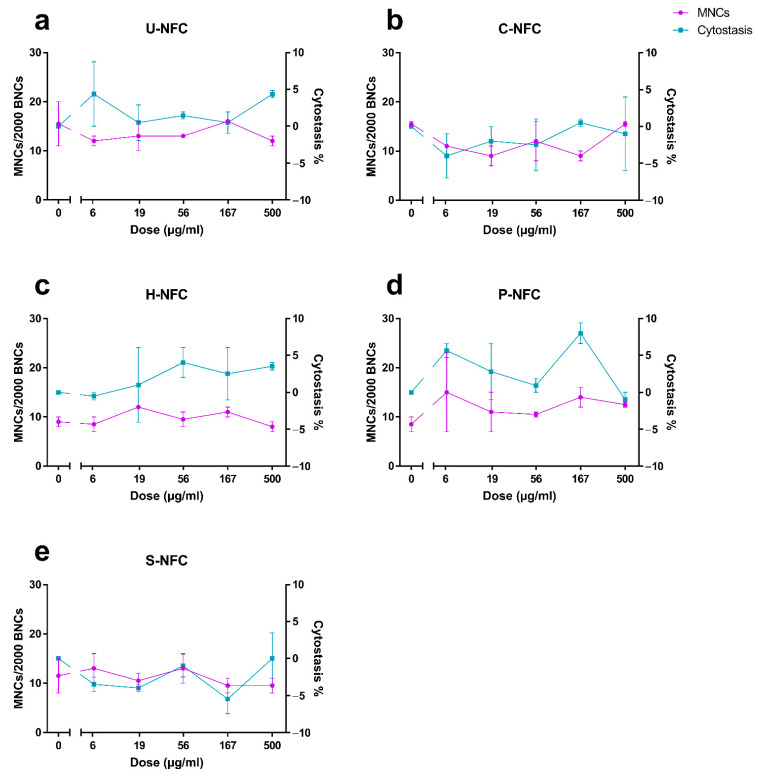
Induction of micronuclei by the nanofibrillated cellulose (NFC) materials. Micronucleus induction was assessed at 48 h exposure to (**a**) U-NFC, (**b**) C-NFC, (**c**) H-NFC, (**d**) P-NFC and (**e**) S-NFC. Purple symbols show the frequency of micronucleated cells in 2000 binucleated cells (MNCs/2000 BNCs). Blue symbols show the percentage of cytostasis. Data are presented as the mean ± standard error of the mean.

As requested by OECD TG 487 [37], cytotoxicity or cytostasis should be assessed in all cultures that are scored for micronuclei, to demonstrate appropriate cell proliferation. The results showed that 55 ± 5% cytostasis, set as the upper limit for testing by the guideline, was exceeded by none of the cellulosic materials, at none of the tested doses (Figure 7). The cytostasis values were <10% for all the NFC materials.

None of the NFC materials were able to induce an increase in chromosome damage compared with the negative control at any of the tested doses (Figure 7).

The positive control, MMC (150 ng/mL), induced a statistically significant increase in the frequency of micronucleated cells over the negative control values in all the experiments performed (22.4 ± 3.4-fold increase; *p* < 0.01; 79.0 ± 1.0% cytostasis), confirming the validity of the experiments.

## 4. Discussion

As NFC is expected to have a wide range of industrial and biomedical applications and thereby an increasing presence in the market, the safety assessment of various forms of the material becomes a priority [10]. The present study aimed at increasing knowledge about the possible interactions of NFC with human lung cells, with a special focus on the effect of surface chemistry. Surface chemical modification of NFC is commonly used as a strategy to facilitate the fibrillation process during production and to endow NFC with distinct properties for different applications [8,9]. By altering the physicochemical properties of nanofibers, functionalization may in turn influence NFC interactions with biological systems. The NFC materials investigated in this study included four different functionalized NFCs (C-NFC, H-NFC, P-NFC and S-NFC) and a nonfunctionalized NFC (U-NFC). Material characterization showed that the introduction of surface charged groups had a marked effect on the morphology of the fibers. The individual nanofibrils observed in the functionalized NFC samples could be a consequence of electrostatic repulsion between the fibers, due to the presence of the surface charged groups [8], while fiber aggregates predominated in U-NFC. Moreover, differences in surface group density among the functionalized NFC materials influenced the extent of fiber aggregation, with thicker fibers being observed for the less substituted C-NFC and S-NFC compared with H-NFC and P-NFC.

Human bronchial epithelial BEAS-2B cells constituted the in vitro model used to investigate cellular response to the NFC materials. This cell line (wild-type for p53), able to be grown in serum-free medium and to enter squamous differentiation, has a normal antioxidant capacity and has been reported to be very similar to primary bronchial cells and to human lung tissue in terms of gene expression pattern [42,43]. Hence, it is assumed to be a good model for human lung research. In addition, BEAS-2B has been suggested to be an appropriate in vitro model in asbestos and nanotoxicological research and is capable of material endocytosis while undergoing cell division [44,45]. However, the NFC materials investigated in the present study did not seem to be efficiently taken up by the BEAS-2B cells, as most cells showed no associated calcofluor-stained NFC. In agreement with these results, NFCs with the same surface modifications (U-NFC, C-NFC and H-NFC) as those assessed in the present study were not internalized by human THP-1 macrophages [25]. Two other types of NFC (freeze-dried powder and 0.9% wt gel) were neither internalized by human lung alveolar epithelial A549 cells treated with 45 µg/cm^2^ of the materials for 72 h [46]. Instead, the NFCs were mostly localized at the cell boundaries. Interestingly, cellulose nanocrystals analyzed in parallel were clearly seen inside the A549 cells [46]. Furthermore, Tomić et al. [35] found that unmodified NFC materials were partially or completely internalized by monocyte-derived dendritic cells, depending on whether they were larger or smaller in size, respectively. In contrast, cellulose nanocrystals were completely internalized by these cells.

No remarkable toxic effects were induced by the NFC materials on the pulmonary BEAS-2B cell line. None of the tested NFC materials significantly affected the number of metabolically active viable cells, the highest reduction (~10% compared to the untreated cultures) being induced by the highest tested dose (500 µg/mL) of U-NFC, C-NFC and H-NFC at 24 h exposure. Our results agree with those reported by Lopes et al. [24] for the same types of surface modified NFC materials and dose range. These authors found no signs of cytotoxicity, as evaluated by metabolic activity and cell membrane integrity, in human intestinal Caco-2 cells exposed to the NFCs for 24 and 48 h. Similarly, no cytotoxic effect was induced by another type of unmodified NFC when Caco-2 cells were exposed up to 48 h [47]. Cytotoxicity was neither seen when predigested NFCs were assessed in an in vitro triculture model where Caco-2 cells were included [7]. Likewise, a previous study with NFCs chemically modified in a similar way as in the present study (U-NFC, C-NFC and H-NFC) reported a lack of cytotoxicity in human dermal fibroblasts, lung cells, and macrophages [25]. Exposure of human dermal fibroblasts and human lung MRC-5 fibroblasts to 50–500 µg/mL of each NFC for 24 h showed similar metabolic activity and cellular membrane damage as the nontreated cells. Human THP-1 differentiated macrophages treated in the same way had a significantly higher metabolic activity but similar membrane integrity as compared with untreated cells [25]. Furthermore, no cytotoxic effects were observed in BEAS-2B cells treated for 24 and 48 h with 9.5–950 µg/mL of four different NFCs (carboxylated, carboxymethylated, and two NFCs without chemical pretreatment) [13]. However, one of these NFC materials (enzymatically pretreated) induced a statistically significant increase in the percentage of dead macrophages after treatment with 100 µg/mL for 3, 6, and 24 h [14]. Other types of wood-derived NFC materials tested in lung cell lines have given contradictory results. The two previously described NFCs analyzed by Menas et al. [46] in A549 cells caused a significant decrease in cell viability at 72 h postexposure. On the other hand, the viability of the same cell line was not affected by freeze-dried powder of hydrophilic and hydrophobic forms of NFCs [48], although both NFCs induced dose-dependent cytotoxic and inflammatory responses in THP-1 cells.

In the present work, the formation of intracellular ROS in BEAS-2B cells was triggered by U-NFC after 24 h and by C-NFC after 3 h, 6 h and 24 h exposure. H-NFC, P-NFC, or S-NFC did not induce a significant formation of intracellular ROS at any of the exposure times. The ROS induction by U-NFC and C-NFC detected in our study may have been triggered by the endotoxins present in the NFC samples. All tested NFC materials tested by us, except H-NFC, showed a high endotoxin level above the upper detection limit. As the endotoxin content of these four NFCs could not be determined accurately, it remains unclear whether the ROS induction could have reflected a substantially higher amount of endotoxin in C-NFC and U-NFC compared with the other NFCs. However, although the involvement of endotoxins in inducing oxidative stress in different cell types cannot be excluded [49], evidence in favor of endotoxin affecting in vitro toxicity endpoints other than immunological responses is limited [50]. It should also be noted that although (1,3)-β-D-glucans, impurities commonly present in cellulose-based materials [51], are known to have a wide range of bioactivities including immunomodulatory properties [40,52], there are no documented effects on the response of BEAS-2B cells to these bioactive molecules. Moreover, all NFC materials under study showed (1,3)-β-D-glucan levels below the limit for glucan contaminants in biomedical products and therefore such levels are not expected to induce a significant biological response [40]. Overall, we found a distinct response in terms of ROS production to the differently functionalized NFCs. However, the contribution of endotoxin contamination to such response cannot be completely dismissed.

In a previous study, U-NFC and C-NFC materials, as well as another one with the same surface modification as the present H-NFC, showed no significant ROS increase in THP-1 macrophages treated for up to 120 min with the same doses [25]. The differences in the ROS formation reported in both studies may be due to differences in the sensitivity of the used cell lines (BEAS-2B cells vs. THP-1 macrophages) or the length of the exposure treatments (minimum of 3 h vs. 120 min). Oxidative stress was induced in human bronchial alveolar A459 cells after 24 h and 72 h exposure of gel and powder forms of NFC [46]. However, no induction of oxidative stress was reported in the immortalized fibroblast cell-line L929 treated with an unmodified NFC for 48 h [53] or in an in vitro triculture model treated with a predigested NFC for 30 min [7].

To date, few studies have investigated the genotoxic potential of NFCs [2,19]. In the present study, none of the tested NFCs induced a statistically significant increase in micronucleated cells. P-NFC was the only material that showed a linear dose-response (*p* = 0.04) in DNA damage. However, as none of the doses significantly differed from the untreated cultures, P-NFC was not considered to be genotoxic. The present results agree with our previous ones using BEAS-2B cells [13]. None of the assessed NFCs (two nonfunctionalized—one of them enzymatically pretreated—carboxylated and carboxymethylated NFCs) were able to induce DNA or chromosomal damage after treating the cells with up to 950 µg/mL of the materials for 24 and 48 h, respectively. However, the enzymatically pretreated NFC induced a significant dose-dependent increase in DNA damage in the lung cells of mice 24 h after administration (up to 18 μg/mouse [15] and 200 µg/mouse [13]). The effect could still be observed 28 days postadministration in one of the studies [13]. On the other hand, the unmodified nonenzymatically pretreated NFC also induced a significant increase in DNA damage at 24 h postadministration, although no dose-dependency was observed [13]. None of the NFCs induced chromosome damage in peripheral blood erythrocytes of mice at any of the postadministration times [13]. The lack of agreement between the in vitro and in vivo results observed in the study of Lindberg et al. [13] suggests that the mechanisms responsible for the effects observed in vivo may not adequately be present in the in vitro cell systems used. One possibility is that the observed in vivo genotoxicity is induced by a secondary mechanism mediated by an inflammatory response [54]. In fact, all NFCs analysed by Lindberg et al. [13] triggered a pulmonary inflammatory response in mice 24 h after treatment (with up to 18 μg/mouse [15] or 40 µg/mouse [14]), although the inflammation subsided within one month. Furthermore, the enzymatically pretreated NFC also induced a significant increase in the proinflammatory cytokines IL-1β and TNF-α in THP-1 cells [14]. Secondary genotoxic effects of nanomaterials could be detected in coculture systems, which may better mimic the in vivo toxicological response as compared with monocultures [55,56]. In fact, a TEMPO oxidized NFC induced a significant increase in chromosome damage, assessed by the micronucleus assay, in lung epithelial alveolar A549 cells cocultured with THP-1 macrophages [27]. No inflammatory response was reported in this system, although the assessment was only based on measuring the production of the proinflammatory cytokine IL-1β. A more sophisticated 3D in vitro triple cell coculture model of the human epithelial airway barrier was used by Clift et al. [57] to assess the cytotoxicity and inflammatory response of cellulose nanowhiskers isolated from cotton. The model consisted of human monocyte derived macrophages, dendritic cells, and human bronchial epithelial 16HBE14o- cells. A dose-dependent cytotoxic and proinflammatory response was observed for the nanowhiskers. However, this system has so far not been used for testing NFC. Considering that a significant release of IL-1β and TNF-α was induced by U-NFC, but not by C-NFC or H-NFC, in THP-1 macrophages treated with up to 500 µg/mL for 24 h [25], it would also be interesting to find out whether U-NFC could induce genotoxic effects by an inflammatory-mediated mechanism, and if the introduction of surface-groups could modulate this effect.

## 5. Conclusions

In conclusion, our findings show that none of the NFC materials tested, including different types of surface functionalization with different surface charges, can induce genotoxic effects in human bronchial epithelial BEAS-2B cells in vitro. The NFCs neither elicited cytotoxic effects. The nonfunctionalized and carboxymethylated NFCs were able to increase intracellular formation of ROS. However, the ROS increase did not result in an increased DNA or chromosome damage.

## Figures and Tables

**Table 1:** Properties of the nanofibrillated cellulose (NFC) materials under study.

				z-Potential (mV)	
NFC Sample	Surface Modification	Functional Group Content (μmol/g)	Fiber Diameter (Aqueous Suspension) ^1^	NaCl (10 mM, pH 7.5) ^2^	Cell Culture Medium LHC-9 (pH 6–8) ^3^
U-NFC	None	30 ^4^	10–30 nm aggregates	−10 ± 2.5 ^5^	−14.1 ± 5.2
C-NFC	Carboxymethylation	371	Some individual fibrils, fiber aggregates (10–15 nm)	−20.9 ± 1.8	−20.8 ± 0.6
H-NFC	Hydroxypropyl- trimethylammonium substitution	634	4–5 nm individual fibrils	17.4 ± 2.2 ^5^	18.7 ± 1.0
P-NFC	Phosphorylation	1109	4–5 nm individual fibrils	−31.1 ± 1.2 ^5^	−29.6 ± 1.1
S-NFC	Sulfoethylation	444	Some individual fibrils, fiber aggregates (10–12 nm)	−23.8 ± 1.6 ^5^	−17.8 ± 0.7

^1^ Estimated by TEM images; ^2^ measurements done at 25 °C, ^3^ measurements done at 37 °C; ^4^ residual carboxyl groups; ^5^ From Lopes et al. [24].

## Supplementary Materials

The following are available online at https://www.mdpi.com/2079-4991/11/2/389/s1, Figure S1: Levels of (1,3)-β-D-glucans in extracts of the NFC materials under study.

## References

[1] Tan K., Heo S., Foo M., Chew I.M., Yoo C. (2019). An insight into nanocellulose as soft condensed matter: Challenge and future prospective toward environmental sustainability. Sci. Total Environ..

[2] Chinga-Carrasco G., Rosendahl J., Catalán J. Nanocellulose-Nanotoxicology, safety aspects and 3D printing. Adv. Exp. Med. Biol..

[3] Klemm D., Schumann D., Kramer F., Heßler N., Hornung M., Schmauder H.-P., Marsch S., Klemm D. (2006). Nanocelluloses as innovative polymers in research and application. Polysaccharides II.

[4] Habibi Y., Lucia L.A., Rojas O.J. (2010). Cellulose Nanocrystals: Chemistry, Self-Assembly, and Applications. Chem. Rev..

[5] Saito T., Nishiyama Y., Putaux J.-L., Vignon M., Isogai A. (2006). Homogeneous Suspensions of Individualized Microfibrils from TEMPO-Catalyzed Oxidation of Native Cellulose. Biomacromolecules.

[6] Fukuzumi H., Saito T., Isogai A. (2013). Influence of TEMPO-oxidized cellulose nanofibril length on film properties. Carbohydr. Polym..

[7] DeLoid G., Cao X., Molina R.M., Silva D.I., Bhattacharya K., Ng K.W., Loo J.S.C., Brain J.D., Demokritou P. (2019). Toxicological effects of ingested nanocellulose in in vitro intestinal epithelium and in vivo rat models. Environ. Sci. Nano.

[8] Rol F., Belgacem M.N., Gandini A., Bras J. (2019). Recent advances in surface-modified cellulose nanofibrils. Prog. Polym. Sci..

[9] Lavoine N., Desloges I., Dufresne A., Bras J. (2012). Microfibrillated cellulose–Its barrier properties and applications in cellulosic materials: A review. Carbohydr. Polym..

[10] Catalán J., Norppa H. (2017). Safety Aspects of Bio-Based Nanomaterials. Bioengineering.

[11] Shatkin J.A., Kim B. (2015). Cellulose nanomaterials: Life cycle risk assessment, and environmental health and safety roadmap. Environ. Sci. Nano.

[12] Stefaniak A.B., Seehra M.S., Fix N.R., Leonard S.S. (2014). Lung biodurability and free radical production of cellulose nanomaterials. Inhal Toxicol..

[13] Lindberg H.K., Catalán J., Aimonen K.J., Wolff H., Wedin I., Nuopponen M., Savolainen K.M., Norppa H. (2017). Evaluation of the genotoxic potential of different types of nanofibrillated celluloses. TechConnect Briefs.

[14] Ilves M., Vilske S., Aimonen K., Lindberg H.K., Pesonen S., Wedin I., Nuopponen M., Vanhala E., Højgaard C., Winther J.R. (2018). Nanofibrillated cellulose causes acute pulmonary inflammation that subsides within a month. Nanotoxicology.

[15] Hadrup N., Knudsen K.B., Berthing T., Wolff H., Bengtson S., Kofoed C., Espersen R., Højgaard C., Winther J.R., Willemoës M. (2019). Pulmonary effects of nanofibrillated celluloses in mice suggest that carboxylation lowers the inflammatory and acute phase responses. Environ. Toxicol. Pharmacol..

[16] Lynch I., Weiss C., Valsami-Jones E. (2014). A strategy for grouping of nanomaterials based on key physico-chemical descriptors as a basis for safer-by-design NMs. Nano Today.

[17] Endes C., Camarero-Espinosa S., Mueller S., Foster E.J., Petri-Fink A., Rothen-Rutishauser B., Weder C., Clift M.J.D. (2016). A critical review of the current knowledge regarding the biological impact of nanocellulose. J. Nanobiotechnol..

[18] Roman M. (2015). Toxicity of Cellulose Nanocrystals: A Review. Ind. Biotechnol..

[19] Ventura C., Pinto F., Lourenço A.F., Ferreira P.J., Louro H., Silva M.J. (2020). On the toxicity of cellulose nanocrystals and nanofibrils in animal and cellular models. Cellulose.

[20] Gasic S., Tomić S., Bekić M. (2020). Immunological aspects of nanocellulose. Immunol. Lett..

[21] Stoudmann N., Schmutz M., Hirsch C., Nowack B., Som C. (2020). Human hazard potential of nanocellulose: Quantitative insights from the literature. Nanotoxicology.

[22] Park E.J., Khaliullin T.O., Shurin M.R., Kisin E.R., Yanamala N., Fadeel B., Chang J., Shvedova A.A. (2018). Fibrous nanocellulose, crystalline nanocellulose, carbon nanotubes, and crocidolite asbestos elicit disparate immune responses upon pharyngeal aspiration in mice. J. Immunotoxicol..

[23] Bitounis D., Pyrgiotakis G., Bousfield D., Demokritou P. (2019). Dispersion preparation, characterization, and dosimetric analysis of cellulose nano-fibrils and nano-crystals: Implications for cellular toxicological studies. NanoImpact.

[24] Lopes V.R., Strømme M., Ferraz N. (2020). In vitro biological impact of nanocellulose fibers on human gut bacteria and gastrointestinal cells. Nanomaterials.

[25] Lopes V.R., Sanchez-Martinez C., Strømme M., Ferraz N. (2017). In vitro biological responses to nanofibrillated cellulose by human dermal, lung and immune cells: Surface chemistry aspect. Part Fibre Toxicol..

[26] De Lima R., Mattoso L.H.C., Feitosa L.O., Maruyama C.R., Barga M.A., Yamawaki P.C., Vieira I.J., Teixeira E.M., Fraceto L.F. (2012). Evaluation of the genotoxicity of cellulose nanofibers. Int. J. Nanomed..

[27] Ventura C., Lourenço A.F., Sousa-Uva A., Ferreira P.J., Silva M.J. (2018). Evaluating the genotoxicity of cellulose nanofibrils in a co-culture of human lung epithelial cells and monocyte-derived macrophages. Toxicol. Lett..

[28] Catalán J., Rydman E., Aimonen K., Hannukainen K.S., Suhonen S., Vanhala E., Moreno C., Meyer V., Perez D.D., Sneck A. (2017). Genotoxic and inflammatory effects of nanofibrillated cellulose in murine lungs. Mutagenesis.

[29] Pääkkö M., Ankerfors M., Kosonen H., Nykänen A., Ahola S., Österberg M., Ruokolainen J., Laine J., Larsson P.T., Ikkala O. (2007). Enzymatic Hydrolysis Combined with Mechanical Shearing and High-Pressure Homogenization for Nanoscale Cellulose Fibrils and Strong Gels. Biomacromolecules.

[30] Hua K., Ålander E., Lindström T., Mihranyan A., Strømme M., Ferraz N. (2015). Surface Chemistry of Nanocellulose Fibers Directs Monocyte/Macrophage Response. Biomacromolecules.

[31] Naderi A., Lindström T., Flodberg G., Sundström J., Junel K., Runebjörk A., Weise C.F., Erlandsson J. (2016). Phosphorylated nanofibrillated cellulose: Production and properties. Nordic Pulp Paper Res. J..

[32] Naderi A., Koschella A., Heinze T., Shih K.-C., Nieh M.-P., Pfeifer A., Chang C.-C., Erlandsson J. (2017). Sulfoethylated nanofibrillated cellulose: Production and properties. Carbohydr. Polym..

[33] Usov I., Nyström G., Adamcik J., Handschin S., Schütz C., Fall A., Bergström L., Mezzenga R. (2015). Understanding nanocellulose chirality and structure–properties relationship at the single fibril level. Nat. Commun..

[34] Reddel R.R., Ke Y., Gerwin B.I., McMenamin M.G., Lechner J.F., Su R.T., Brash D.E., Park J.-B., Rhim J.S., Harris C.C. (1988). Transformation of Human Bronchial Epithelial Cells by Infection with SV40 or Adenovirus-12 SV40 Hybrid Virus, or Transfection via Strontium Phosphate Coprecipitation with a Plasmid Containing SV40 Early Region Genes. Cancer Res..

[35] Tomić S., Kokol V., Mihajlović D., Mirčić A., Gasic S. (2016). Native cellulose nanofibrills induce immune tolerance in vitro by acting on dendritic cells. Sci. Rep..

[36] Guadagnini R., Halamoda Kenzaoui B., Walker L., Pojana G., Magdolenova Z., Bilanicova D., Saunders M., Juillerat-Jeanneret L., Marcomini A., Huk A. (2015). Toxicity screenings of nanomaterials: Challenges due to interference with assay processes and components of classic in vitro tests. Nanotoxicology.

[37] OECD (2016). Test No. 487: In Vitro Mammalian Cell Micronucleus Test.

[38] Vales G., Suhonen S., Siivola K.M., Savolainen K.M., Catalán J., Norppa H. (2020). Size, Surface Functionalization, and Genotoxicity of Gold Nanoparticles In Vitro. Nanomaterials.

[39] FDA (US Food and Drug Administration) (2014). Bacterial Endotoxins/Pyrogens. https://www.fda.gov/inspections-compliance-enforcement-and-criminal-investigations/inspection-technical-guides/bacterial-endotoxinspyrogens.

[40] Barton C., Vigor K., Scott R., Jones P., Lentfer H., Bax H.J., Josephs D.H., Karagiannis S.N., Spicer J.F. (2016). Beta-glucan contamination of pharmaceutical products: How much should we accept?. Cancer Immunol. Immunother..

[41] Galloway S.M. (2000). Cytotoxicity and chromosome aberrations in vitro: Experience in industry and the case for an upper limit on toxicity in the aberration assay. Environ. Mol. Mutagenesis.

[42] Kinnula V.L., Yankaskas J.R., Chang L., Virtanen I., Linnala A., Kang B.H., Crapo J.D. (1994). Primary and immortalized (BEAS 2B) human bronchial epithelial cells have significant antioxidative capacity in vitro. Am. J. Respir. Cell Mol. Biol..

[43] Garcia-Canton C., Minet E., Anadon A., Meredith C. (2013). Metabolic characterization of cell systems used in in vitro toxicology testing: Lung cell system BEAS-2B as a working example. Toxicol. In Vitro.

[44] Haniu H., Saito N., Matsuda Y., Kim Y.-A., Park K.C., Tsukahara T., Usui Y., Aoki K., Shimizu M., Ogihara N. (2011). Elucidation mechanism of different biological responses to multi-walled carbon nanotubes using four cell lines. Int. J. Nanomed..

[45] Nymark P., Wijshoff P., Cavill R., van Herwijnen M., Coonen M.L.J., Claessen S., Catalán J., Norppa H., Kleinjans J.C.S., Briedé J.J. (2015). Extensive temporal transcriptome and microRNA analyses identify molecular mechanisms underlying mitochondrial dysfunction induced by multi-walled carbon nanotubes in human lung cells. Nanotoxicology.

[46] Menas A.L., Yanamala N., Farcas M.T., Russo M., Friend S., Fournier P.M., Star A., Iavicoli I., Shurin G.V., Vogel U.B. (2017). Fibrillar vs. crystalline nanocellulose pulmonary epithelial cell responses: Cytotoxicity or inflammation?. Chemosphere.

[47] Chen Y., Lin Y.-J., Nagy T., Kong F., Guo T.L. (2020). Subchronic exposure to cellulose nanofibrils induces nutritional risk by non-specifically reducing the intestinal absorption. Carbohydr. Polym..

[48] Yanamala N., Kisin E.R., Menas A.L., Farcas M.T., Khaliullin T.O., Vogel U.B., Shurin G.V., Schwegler-Berry D., Fournier P.M., Star A. (2016). In Vitro Toxicity Evaluation of Lignin-(Un)coated Cellulose Based Nanomaterials on Human A549 and THP-1 Cells. Biomacromolecules.

[49] Fernández-Cruz M.L., Hernández-Moreno D., Catalán J., Cross R.K., Stockmann-Juvala H., Cabellos J., Lopes V.R., Matzke M., Ferraz N., Izquierdo J.J. (2018). Quality evaluation of human and environmental toxicity studies performed with nanomaterials–the GUIDEnano approach. Environ. Sci. Nano.

[50] Pagani R., Portolés M.T., Díaz-Laviada I., Municio A.M. (1988). Morphological damage induced by Escherichia coli lipopolysaccharide in cultured hepatocytes: Localization and binding properties. Br. J. Exp. Pathol..

[51] Liu J., Bacher M., Rosenau T., Willför S., Mihranyan A. (2018). Potentially Immunogenic Contaminants in Wood-Based and Bacterial Nanocellulose: Assessment of Endotoxin and (1,3)-β-d-Glucan Levels. Biomacromolecules.

[52] Barsanti L., Passarelli V., Evangelista V., Frassanito A.M., Gualtieri P. (2011). Chemistry, physico-chemistry and applications linked to biological activities of β-glucans. Nat. Prod. Rep..

[53] Čolić M., Mihajlović D., Mathew A.P., Naseri N., Kokol V. (2015). Cytocompatibility and immunomodulatory properties of wood based nanofibrillated cellulose. Cellulose.

[54] Evans S.J., Clift M.J.D., Singh N., de Oliveira Mallia J., Burgum M., Wills J.W., Wilkinson T.S., Jenkins G.J.S., Doak S.H. (2017). Critical review of the current and future challenges associated with advanced in vitro systems towards the study of nanoparticle (secondary) genotoxicity. Mutagenesis.

[55] Snyder-Talkington B.N., Qian Y., Castranova V., Guo N.L. (2012). New Perspectives for in Vitro Risk Assessment of Multiwalled Carbon Nanotubes: Application of Coculture and Bioinformatics. J. Toxicol. Environ. Health Part B.

[56] Snyder-Talkington B.N., Dong C., Zhao X., Dymacek J., Porter D.W., Wolfarth M.G., Castranova V., Qian Y., Guo N.L. (2015). Multi-walled carbon nanotube-induced gene expression in vitro: Concordance with in vivo studies. Toxicology.

[57] Clift M.J.D., Foster E.J., Vanhecke D., Studer D., Wick P., Gehr P., Rothen-Rutishauser B., Weder C. (2011). Investigating the interaction of cellulose nanofibers derived from cotton with a sophisticated 3D human lung cell coculture. Biomacromolecules.

