# Supplementary material for: Role of Surface Chemistry in the In Vitro Lung Response to Nanofibrillated Cellulose
Source: Nanomaterials (Basel). 2021 Feb 3;11(2):389. doi: 10.3390/nano11020389 (PMC7913598; doi:10.3390/nano11020389)
Supplement: Supplementary file 1 [file nanomaterials-11-00389-s001.pdf]

Supplementary Information

Genotoxicity of nanofibrillated celluloses with different surface chemistry

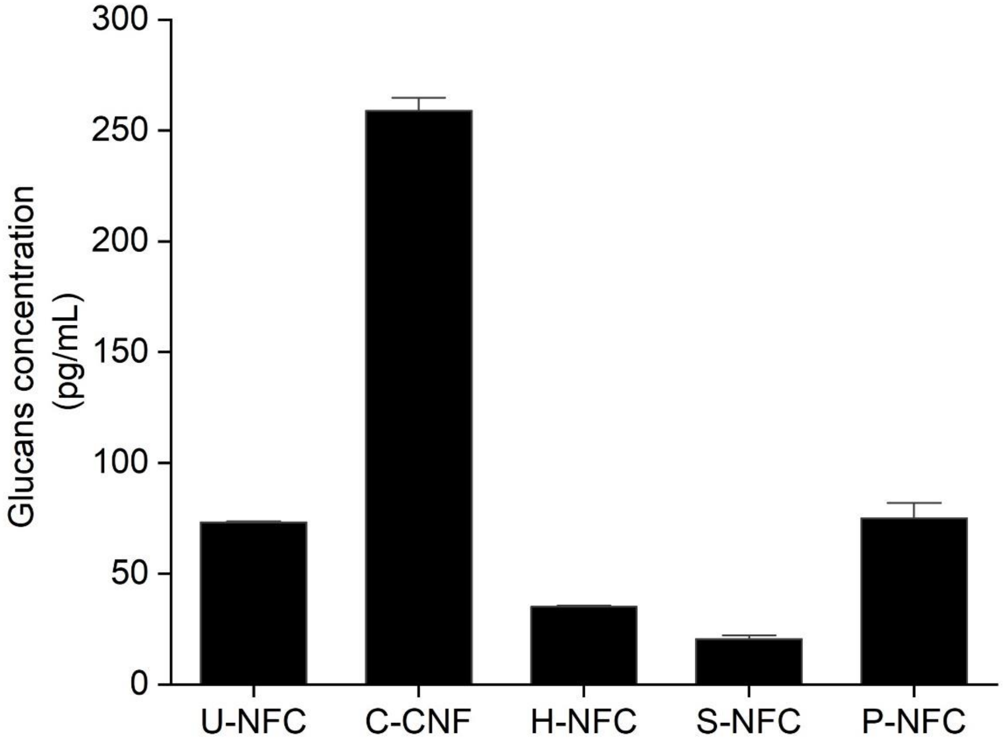

Figure S1. Levels of (1,3)-β-D-glucans in extracts of the NFC materials under study.
